# Estimation of the burden of cardiovascular disease attributable to modifiable risk factors and cost-effectiveness analysis of preventative interventions to reduce this burden in Argentina

**DOI:** 10.1186/1471-2458-10-627

**Published:** 2010-10-20

**Authors:** Adolfo Rubinstein, Lisandro Colantonio, Ariel Bardach, Joaquín Caporale, Sebastián García Martí, Karin Kopitowski, Andrea Alcaraz, Luz Gibbons, Federico Augustovski, Andrés Pichón-Rivière

**Affiliations:** 1 Institute for Clinical Effectiveness and Health Policy (IECS), Buenos Aires, Argentina; 2 Division of Family and Community Medicine, Hospital Italiano de Buenos Aires, Buenos Aires, Argentina; 3 Programa de Prevención del Infarto en Argentina (PROPIA), Universidad Nacional de La Plata, Buenos Aires, Argentina; 4 Centro de Endocrinología Experimental y Aplicada (CENEXA), Universidad Nacional de La Plata, Buenos Aires, Argentina

## Abstract

**Background:**

Cardiovascular disease (CVD) is the primary cause of mortality and morbidity in Argentina representing 34.2% of deaths and 12.6% of potential years of life lost (PYLL). The aim of the study was to estimate the burden of acute coronary heart disease (CHD) and stroke and the cost-effectiveness of preventative population-based and clinical interventions.

**Methods:**

An epidemiological model was built incorporating prevalence and distribution of high blood pressure, high cholesterol, hyperglycemia, overweight and obesity, smoking, and physical inactivity, obtained from the Argentine Survey of Risk Factors dataset. Population Attributable Fraction (PAF) of each risk factor was estimated using relative risks from international sources. Total fatal and non-fatal events, PYLL and Disability Adjusted Life Years (DALY) were estimated. Costs of event were calculated from local utilization databases and expressed in international dollars (I$). Incremental cost-effectiveness ratios (ICER) were estimated for six interventions: reducing salt in bread, mass media campaign to promote tobacco cessation, pharmacological therapy of high blood pressure, pharmacological therapy of high cholesterol, tobacco cessation therapy with bupropion, and a multidrug strategy for people with an estimated absolute risk > 20% in 10 years.

**Results:**

An estimated total of 611,635 DALY was lost due to acute CHD and stroke for 2005. Modifiable risk factors explained 71.1% of DALY and more than 80% of events. Two interventions were cost-saving: lowering salt intake in the population through reducing salt in bread and multidrug therapy targeted to persons with an absolute risk above 20% in 10 years; three interventions had very acceptable ICERs: drug therapy for high blood pressure in hypertensive patients not yet undergoing treatment (I$ 2,908 per DALY saved), mass media campaign to promote tobacco cessation amongst smokers (I$ 3,186 per DALY saved), and lowering cholesterol with statin drug therapy (I$ 14,432 per DALY saved); and one intervention was not found to be cost-effective: tobacco cessation with bupropion (I$ 59,433 per DALY saved)

**Conclusions:**

Most of the interventions selected were cost-saving or very cost-effective. This study aims to inform policy makers on resource-allocation decisions to reduce the burden of CVD in Argentina.

## Background

Chronic diseases are increasing in developing countries and cardiovascular diseases account for 17.7 million annual deaths around the world, constituting 11% of estimates for the global burden of disease. It is estimated that mortality due to coronary heart disease (CHD) and stroke will increase by approximately 145% among men and women from 1990 to 2020 in Latin America, compared with a 28% increase for women and a 50% increase for men over the same period in developed countries [1].

In Argentina, chronic non-communicable diseases account for more than 50% of the overall morbidity and mortality. In fact, the age-adjusted mortality rate of cardiovascular disease, including CHD and stroke was 206.4 per 100,000 (265.4 for men and 161.8 for women), representing 34.2% of deaths and 12.6% of years of potential life lost [2]. Adjusted mortality rates for non-communicable diseases, as well as Potential Years of Life Lost (PYLL) have declined steadily since 1987, while mortality rates of communicable, maternal, perinatal and nutritional conditions have remained relatively constant in the same 20-year period. Still, the adjusted rate for non-communicable chronic diseases has been five to six times the rate of communicable diseases in Argentina, and the absolute number of deaths is increasing due to the increasingly elderly population [3].

In common with many other Latin American countries, Argentina falls into an intermediate mortality group where the main risk factors for disease are hypertension, an elevated body mass index (BMI), alcohol abuse and smoking [4]. Elevated BMI is due to excess calories and insufficient activity, and a large proportion of hypertension is due to these same lifestyle risks in addition to a poor diet quality. Primary data describing the prevalence and distribution of cardiovascular risk factors in Argentina has recently been obtained through two different population-based sources: the 2005 Ministry of Health National Risk Factor Survey [5]; and the Cardiovascular Risk Factor Multiple Evaluation in Latin America (CARMELA) [6]. There is strong evidence that a 50% reduction in cardiovascular deaths can be attributable to the reduction of just three modifiable risk factors, namely tobacco use, high blood pressure and elevated cholesterol [7]. In Latin America, the majority of cardiovascular risk could be explained by tobacco use, abnormal lipids, abdominal obesity and high blood pressure as shown in the recently published INTERHEART Latin American study [8]. Most cardiovascular diseases are preventable and there is evidence that supports the effectiveness of interventions to reduce the burden of cardiovascular disease through strategies that reduce risk factors. Unfortunately, strategies to manage cardiovascular conditions have been largely developed for high-income countries which may not be affordable to most of the developing world [9,10]. Although there has been widespread recognition of the benefit of cost-effectiveness evaluation to inform national health systems of priority settings, its potential has not been realized in the vast majority of countries, including Argentina [11]. Nevertheless, cost-effective interventions to prevent cardiovascular disease in developing countries do exist, but have not been widely applied. Specifically, population and community-based interventions appear to be highly cost-effective when they reach large populations, address high mortality and morbidity diseases, and include multi-level integrated efforts. Interventions targeting individuals, especially high cardiovascular risk subjects, are also cost-effective but usually require clinical involvement and more resources. Moreover, recent studies have consistently shown the cost-effectiveness of interventions that lower the burden of cardiovascular disease in developing countries [12-14].

The aims of this study were 1) to develop an analytical model to estimate the burden of acute CHD and stroke attributable to modifiable cardiovascular disease risk factors in Argentina, 2) to explore the costs of major cardiovascular events, and 3) to calculate the cost-effectiveness of different population-based and clinical interventions in order to inform local policy makers on resource-allocation priority setting.

## Methods

We conducted a population-level comparative risk assessment for seven modifiable cardiovascular risk factors to be included in a model to assess their impact on major cardiovascular events: acute myocardial infarction (AMI), other non-infarction ischemic events and stroke. We also estimated the individual and aggregate effect of population-based and clinical interventions that might modify the risks associated to these risk factors. These interventions are supported by evidence in the literature for clinical efficacy and population effectiveness estimates that take into account detection and patient compliance. Cardiovascular risk factors and interventions were modeled for the adult population over 35 years old since they are the usual target for most clinical interventions. Finally, cardiovascular events, Disability Adjusted Life Years (DALY) and interventions costs were derived.

### Selection of Risk Factors

We selected specific risk factors that fulfilled the following criteria: (1) Sufficient evidence was available on the presence and magnitude of likely causal association with CHD and stroke from high-quality epidemiological studies, (2) available interventions existed to modify associated risk, (3) data on risk factor prevalence was available from the First Argentinean Survey of Risk Factors (FASRF) or other nationally representative surveys not subjected to selection bias.

The seven modifiable risk factors selected were: 1) high blood pressure (HBP), 2) high cholesterol, 3) overweight and obesity, 4) elevated fasting glucose level and type-2 diabetes mellitus, 4) tobacco smoking, 5) physical inactivity,

Unfortunately, consumption of vegetables and fruits was ill-defined in the FASRF since the daily quantity of servings was not specified, for which we had to exclude this measure for further analysis. Other specific individual dietary factors that would meet criteria for causal effects, such as intake of trans fat, low marine omega-3 (seafood), and low polyunsaturated fat (exchanged for saturated fat) were also excluded because of lack of reliable data on their respective prevalences, after a thorough search of local surveys.

### Data Sources

#### Risk Factors exposure

We obtained risk factor prevalence and distribution for each individual enrolled in the FASRF is a nationally representative survey including 41,393 subjects from all districts of the country sampled through a probabilistic multi-stage process [5]. The prevalence of risk factors was obtained from self-reports obtained during an in-person interview that was subsequently validated with direct measures in one district. For those subjects who reported not to have ever measured her/his blood pressure (11.94%), serum cholesterol (43.25%) or glycemia (23.49%) we considered them as not having the risk factor in the survey. As this assumption could have underestimated their prevalence and population-attributable risk, we developed a logistic regression model to estimate the odds and probabilities for a subject with a certain demographic and risk factor profile to have an abnormal value in each of these three risk factors. These new set of risk factors prevalence were used as an alternative scenario in the sensitivity analysis. STATA v8.0 was used to run these models.

#### Etiological effects of risk factors on disease-specific mortality

We obtained the relative risk for CHD and stroke attributable to each risk factor for each exposure category (since all risks were measured in categories in the FASRF), based on published observational studies, systematic reviews or meta-analyses of epidemiological studies. In previous observational studies used for effect sizes, the majority had adjusted for potential confounding factors. Each relative risk used in our analysis represents the best judgment of the evidence for the effect of risk factor exposure on disease-specific mortality. The etiological effect sizes along different age-strata and gender are shown in Table 1.

**Table 1:** List of relative risks included into the model

	Age groups							
	18-39	40-44	45-59	60-64	65-69	70-79	80+	Reference
**Relative risk for coronary heart disease**								
High blood pressure (m)	1.3	2.0	2.2	2.7	2.7	2.7	2.2	[83-85]
High blood pressure (w)	2.5	2.5	2.2	2.7	2.7	2.7	2.2	[83-85]
High glycemia (m)	2.0	2.0	2.0	2.0	2.0	2.0	2.0	[86]
High glycemia (w)	2.5	2.5	2.5	2.5	2.5	2.5	2.5	[86]
Overweight (m)	1.2	1.2	1.2	1.2	1.2	1.2	1.2	[87]
Overweight (w)	1.2	1.2	1.2	1.2	1.2	1.2	1.2	[87]
Obesity (m)	1.5	3.0	3.0	3.0	1.5	1.5	1.5	[87]
Obesity (w)	1.5	1.5	1.5	1.5	1.5	1.5	1.5	[87]
High cholesterol (m)	2.0	2.0	2.0	2.0	2.0	2.0	2.0	[8]
High cholesterol (w)	3.4	3.4	3.4	3.4	3.4	3.4	3.4	[8]
Current smoker (m)	2.4	2.4	2.4	1.8	1.8	1.7	1.4	[88]
Current smoker (w)	2.2	2.2	2.4	2.1	2.1	1.7	1.3	[88]
Former smoker (m)	1.3	1.3	1.3	1.3	1.2	1.2	1.2	[89]
Former smoker (w)	1.6	1.6	1.6	1.6	1.2	1.2	1.2	[89]
								
								
Non-sedentary life style (m)	0.7	0.7	0.7	0.7	0.7	0.7	0.7	[8]
Non-sedentary life style (w)	0.7	0.7	0.7	0.7	0.7	0.7	0.7	[8]
**Relative risk for stroke**								
High blood pressure (m)	3.0	3.0	2.9	2.4	2.4	2.1	1.5	[83]
High blood pressure (w)	2.4	2.4	2.2	2.1	2.1	1.9	1.5	[83]
High glycemia (m)	2.0	2.0	2.0	2.0	2.0	2.0	2.0	[86]
High glycemia (w)	2.0	2.0	2.0	2.0	2.0	2.0	2.0	[86]
Overweight (m)	1.2	1.2	1.2	1.2	1.2	1.2	1.2	[87]
Overweight (w)	1.1	1.1	1.1	1.1	1.1	1.1	1.1	[87]
Obesity (m)	1.6	1.6	1.6	1.6	1.6	1.6	1.6	[90]
Obesity (w)	1.3	1.3	1.3	1.3	1.3	1.3	1.3	[90]
High cholesterol (m)	1.2	1.2	1.2	1.2	1.2	1.2	1.2	[91]
High cholesterol (w)	1.2	1.2	1.2	1.2	1.2	1.2	1.2	[91]
Current smoker (m)	2.4	2.4	2.4	1.8	1.8	1.7	1.4	[88]
Current smoker (w)	2.2	2.2	2.2	2.1	2.1	1.7	1.3	[88]
Former smoker (m)	1.0	1.0	1.0	1.0	1.0	1.0	1.0	[89]
Former smoker (w)	1.3	1.3	1.3	1.3	1.0	1.0	1.0	[89]
								
								
Non-sedentary life style (m)	0.8	0.8	0.8	0.8	0.8	0.8	0.8	[92]
Non-sedentary life style (w)	0.8	0.8	0.8	0.8	0.8	0.8	0.8	[92]

m: men; w: women.

#### Disease-specific deaths

The number of deaths by CHD (ICD-10 codes I20×, I24× and I25× for non-infarction ischemic events and I21× and I22× for AMI) and stroke (ICD-10 codes I60-I61, I63-I64) were obtained from the National Directorate of Health Statistics of the Argentine Ministry of Health [15].

### Estimating mortality and disability attributable to risk factors

For each risk factor and for each disease causally associated with its exposure, we computed the proportional reduction in disease-specific deaths that would occur if risk factor prevalence had been reduced to zero. This is known as the population-attributable risk (PAR) and measures the total effects of a risk factor (direct as well as mediated through other factors). In order to estimate the PAR of each risk factor, we developed an epidemiological simulation model in Microsoft Excel(r), containing the prevalence and distribution of risk factors according to each age and sex strata as observed in the FASRF [5]. In this way, this matrix of 41,392 registries from the FASRF, representing the Argentine population, was split into all possible combinations of risk factors. Additional risk for each combination was assumed to be the product (multiplication) of the relative risk of the risk factors involved [16]. Finally, the baseline absolute risks for both fatal and non-fatal events for people without any of the selected risk factors were derived considering the overall risk, prevalence and additional risk associated to each combination. The global risk of death (across all combinations and age groups) was calibrated against the overall number of deaths due to CHD and stroke corresponding to Argentina in the year 2005 [15]. Finally, the number of non-fatal events for each death from CHD and stroke was extrapolated using the lethality rate from the Public Hospital registry corresponding to the year 2000 [17].

In addition to the estimation of the prevalence of cardiovascular risk factors and their associated relative risk, the spreadsheet contained the cost and disutility associated with each event in order to obtain a deterministic estimate of the burden of disease, expressed in DALY and overall costs. A DALY is a summary measure that combines years of life lost due to premature death and years of life lived with disability [18]. One DALY can be thought of as one lost year of healthy life. DALYs were calculated based on the model developed by Murray et al. [9].

The duration of disability was estimated by using the software DISMOD II [19]. Disability weights were obtained from two Australian studies on burden of disease [20,21]. For the calculation of years of life lost due to premature death, we used a life expectancy at birth of 80 and 82.5 for men and women, respectively, as recommended for global comparisons in the Global Burden of Disease study [9]. Finally, years of life lost due to premature death were obtained from National death registries and years of life lived with disability were obtained by multiplying the estimated number of non-fatal events by each disability weight, for each age gender strata [19].

In order to estimate the PAR associated to each risk factor, a new estimation of deaths, non fatal events, DALY and costs of CHD and stroke were calculated. These estimations were obtained multiplying the basal absolute risk by the product of the relative risks involved in each combination stratum, assuming a relative risk equal to 1 for the index risk factor, weighted by its respective prevalence. Overall deaths, non fatal events, DALY and costs between the estimation for Argentina in 2005 and the new estimation without the index risk factor, was assumed to be the PAR attributable to that particular risk factor. We programmed a macro using Python language [22], in which we performed 1,000 iterations of the prevalence for each combination of risk factors assuming a binomial distribution. Therefore, a new absolute risk was obtained in each iteration, and new estimations of total deaths, non fatal events, DALY and costs were obtained. Finally, we used the empirical PAR distribution to estimate the 95% confidence interval (95%CI) using the percentile method.

### Definition and Selection of Interventions

Different population-based and clinical interventions to reduce cardiovascular disease burden were explored considering not only the evidence of efficacy and effectiveness [23] but also the feasibility to be implemented in Argentina. Relative risk reductions of the interventions were adjusted by population effectiveness measures taking into account target population coverage as well as patient compliance. All interventions have a time horizon of 5 years after which maximum population effectiveness is assumed. The evidence about population effectiveness of mass media campaign targeted to the promotion of physical activity [24-27], salt reduction in food [28,29], control of overweight and obesity [30-35], and promotion of healthy habits [36,37] was non-conclusive, and hence these interventions were not included in the model. On the other hand, evidence on the effectiveness of media campaigns against smoking was generally strong and local programs had already been implemented [38-42]. Efficacy of interventions were modeled as a relative risk reduction or by a reduction on risk factor prevalence. Effect sizes and joint effect of interventions used in the analysis were based on systematic reviews of randomized trials and meta-analysis, when possible. Intervention effects with their corresponding relative risks estimates are shown in Table 2.

**Table 2:** Effectiveness of selected interventions

Intervention	Efficacy	References
*Population based interventions*		
Mass Media Campaign promote tobacco cessation	Reduction of current smoker prevalence: 7%	[38-42,45]
Reducing salt in bread	RR: 0.99	[28,29]
*Clinical interventions*		
Bupropion treatment for tobacco cessation	Annual cessation rate: 28%.	[49,50]
Pharmacological high blood pressure treatment*	For CHD: RR = 0.66 For stroke: RR = 0.51	[13]
Pharmacological high cholesterol treatment with atorvastatin	For CHD: RR = 0.77 For stroke: RR = 0.81	[13,55]
Treatment with four drugs (Polypill strategy) for people with an absolute cardiovascular risk of more than 20% at 10 years	For CHD: RR = 0.34 For stroke: RR = 0.32	[12,13,53-55]

CHD: Coronary Heart Disease, RR: relative risk.

* Include: atenolol, enalapril, amlodipine and hidroclorothiazide

** Include: aspirine, enalapril, amlodipine and atorvastatin.

#### Population based-interventions

##### Lowering salt intake in the population through reducing salt in bread

A program involving the cooperation between the Government, consumer associations and the Bakery Chambers in an effort to reduce 1 gram of salt per 100 grams of bread. Argentina has an average individual consumption of 12 grams of salt per day, 3.4 grams coming from bread. Local experiences showed that it is possible to reduce the amount of salt in bread without being detected as less palatable. At present, there is a pilot training program implemented in selected cities in Argentina to make bakers reduce salt in bread by using special salt dispensers [43]. This intervention could imply a population-wide reduction of 1.33 mmHg of systolic blood pressure per person and 1% of the PAR of CHD and stroke [28,29,44].

##### Mass Media Campaign to promote tobacco cessation

This program of the National Ministry of Health involves an annual campaign through four TV spots, six radio spots and written material in major newspapers, magazines and public spaces. Costs were retrieved from data from previous campaigns of the National Ministry of Health. This intervention would reduce the prevalence of smoking by 7% [38-42,45].

#### Individual (clinical) interventions

##### Treatment of high blood pressure

Interventions involved lifestyle change promotion and pharmacological therapy to achieve blood pressure control (SBP/DBP less than 140/90). Target population was composed of adults over 35 years old with the diagnosis of high blood pressure and no treatment (over 1.3 millions of Argentine population representing 8.2% people older than 35 years old), estimating for this intervention a relative risk reduction of 44% for CHD and 49% for stroke [13]. We assumed that 40% of the population would take one drug, 40% two drugs and 20% three or more drugs. The drugs and daily doses evaluated were hydrochlorothiazide (25 mg), atenolol (50 mg), enalapril (10 mg), and amlodipine (10 mg), and the treatment mix was 50% of the population taking thiazides, 20% atenolol, 20% angiotensin-converting enzyme inhibitor and 10% amlodipine [46]. The same efficacy for each drug category was also assumed. Analysis indicated that these interventions, with a 50% rate of disease detection and 50% drug compliance as indicated by the Canadian Hypertension Guidelines [47], would reduce PAR of cardiovascular disease and stroke by 8%.

##### Treatment of high cholesterol

This intervention involved promotion of low-cholesterol diet and use of statins (atorvastatin 10 mg, 20 mg and 40 mg for 50%, 40% and 10% of the target population, respectively), according to local estimates and assumptions. Target population was adults over 35 years old with high cholesterol and no treatment (almost one million people representing 5.2% of people older than 35 years old). Achieving a cholesterol target of less than 240 mg/dl, (6.2 mm/l) would provide an estimated reduction of 8% of the PAR of CHD and stroke with a 50% detection and 50% drug compliance rate according to ATP III [48].

##### Tobacco cessation therapy

Motivational interventions from health professionals and drug therapy with bupropion for a 2-month period (300 mg per day) would result in an estimated reduction of 4% of the PAR of CHD and stroke [49]. In most studies with bupropion for tobacco cessation, the annual quitting rate of smokers was 28% vs. 12%, as compared to placebo [50,51]. According to a recent national survey of tobacco prevalence, only 11% of total smokers in Argentina were willing to quit smoking and therefore were considered the target population for this intervention [52]. According to these estimates, the spontaneous annual cessation rate would be 1.32% (12% of the 11% of smokers willing to quit) that would raise to 3.08% with bupropion (28% × 11%), since we would expect a prevalence reduction of 1.76% (3.08%-1.32%).

##### Treatment based on a population absolute risk approach (Polypill strategy)

Since the "Polypill" is not yet in Argentine markets, we designed a pharmacological therapy with 4 pills (hydrochlorothiazide 25 mg, enalapril 10 mg, atorvastatin 10 mg and aspirin 100 mg), prescribed to people older than 35 years old with an estimated combined risk of a cardiovascular event over the next decade above 20%, based on the data from the FASRF. This intervention would imply a relative risk reduction of CHD of 66% (RR = 0.34) and of stroke of 68% (RR = 0.32) [12,13,53-55].

Assuming that at least 50% of the target population is reached by the intervention, a 50% patient compliance rate with treatment for this group, and 70% of provider compliance due to a presumed raised awareness of risks for both subjects, the Polypill strategy would result in a population effectiveness of 17,5%. Relative risks for CHD and stroke for individuals from this high-risk subgroup were estimated by using the beta coefficients from the Framingham Heart Study [16].

### Estimating costs of acute cardiovascular events and interventions

#### Costs of acute events

Cost categories (i.e. inpatient hospital days, doctor visits, tests, drugs and ancillary services, and diagnostic and therapeutic procedures) for AMI, other non-infarction ischemic events such as unstable angina and stroke were first identified. For each event the quantities and unit prices of inputs were retrieved from hospital databases and other local sources [56-66], as well as expert opinion when necessary. The quantities of each input identified were assessed and multiplied by the unit price of each item to obtain the unit cost of each resource. Finally, the total cost of the acute event resulted from the addition of all of the identified consumed resources in each category.

#### Costs of interventions

Costs included program-level expenses associated with management of the interventions (i.e. administration, training and information, dissemination by multiple media sources) and patient-level costs (i.e. primary care visits, ancillary tests and drugs). The quantities of each input required were assessed and multiplied by the unit price of each input for the 5 year intervention implementation period. The quantity of patient-level resource inputs for each intervention (i.e. inpatient hospital days, doctor visits, tests, drugs) were identified from local or international published data if available or expert opinion should the former not be available. Costs of drugs were calculated using a mix of blood pressure lowering drugs composed of 50% hydrochlorothiazide, 20% atenolol, 20% enalapril and 10% amlodipine, according to a published local study [46]. Cost of blood pressure lowering drugs, atorvastatin and bupropion as well as other input costs and expense data were extracted from local sources [65-67]. Other cost data were obtained from the Health Care Costs Database from the Institute of Clinical Effectiveness and Health Policy [62]. A list of costs and sources of the interventions and selected health events is depicted in Table 3.

**Table 3:** Interventions and related health events summary costs

Event cost per hospital admission	2007 I$
Coronary Heart Disease	4,245.39
Stroke	3,455.48
**Population-based interventions**	
Mass Media Campaign promote tobacco cessation*	3,164,785.75
Reducing salt in bread^†^	193,576.23
**Individual interventions (yearly cost per person‡)**	
Pharmacological high blood pressure treatment	49.72
Pharmacological high cholesterol treatment	118.79
Bupropion treatment for tobacco cessation	117,15
Modified Polypill strategy	103.46

I$: international dollars. PPP conversion rate (2007) 1.55 Argentinean peso = 1 I$,

* Ten years duration of campaign, with discounting (3% annual rate).

† Assuming 54 meeting of 30 bakers each (around 800-600 bakers), with discounting (3% annual rate).

‡ Includes health center visits, drug and lab test costs. Programmatic costs were not included (I$ 1,194,067.52).

Note: Cost of blood pressure lowering drugs, atorvastatin and bupropion as well as other input costs and charges data were extracted from local sources [65-67]. Other cost data were obtained from the Health Care Costs Database by the Institute of Clinical Effectiveness and Health Policy [62].

Cost of clinical interventions included, in addition to their specific costs of visits, tests and drugs, 290 countrywide training workshops on cardiovascular risk detection, assessment and control targeted to 8,639 general practitioners from the public and private health sector, along the 5 year period of the intervention, with periodic boosters through email and postal mail. Except when explicitly stated, costs related to labor, equipment, capital, overhead or joint costs were regarded as existing, ongoing, or common to all interventions and therefore were excluded in the calculation. We also excluded costs of accessing health interventions that would include the resources used by patients and their families to obtain an intervention (transport costs) as well as productivity gains or losses, as the study was conducted from a purchaser perspective. All costs were calculated in Argentine pesos for the year 2007, requiring in some cases the use of Health and General CPI to adjust for annual inflation, and finally converted and expressed into international dollars using the Purchase Power Parity conversion rate AR$ 1.55 = 1 I$ [68]. The discounting of long term costs was performed at a 3% rate.

#### Perspective

Since Argentina's healthcare system consists of a multi-tier system divided in three large sectors: public, social security and private, we incorporated the perspective of the whole Argentine healthcare system as a purchaser of health services.

### Calculating cost-effectiveness of interventions

Cost-effectiveness analysis considers the costs and effects of adding new interventions to current practice or the cost of replacing an existing intervention with another targeting the same condition. In order to estimate the reduction in disease burden related to the reduction of cardiovascular disease, we built a model to predict the burden associated with specific diseases or risk factors to develop disease. We calculated the effect of interventions in our model, assuming that all reduced the relative risk associated with the presence of each cardiovascular risk factor. In the case the effect of the intervention was a reduction of the prevalence (i.e.: tobacco cessation), a new relative risk was estimated as a proportional combination of the relative risk associated with the risk factor (for the proportion of people that were still smokers) and the relative risk of those that no longer had that risk factor (i.e.: former smokers). Finally, the model translated these changes into a new estimation of cardiovascular events, overall costs and DALY lost, specific for age and sex. This estimation was then compared to the estimation without the intervention. In addition, the annual cost of the intervention was imputed for the year analyzed. For each intervention, the Incremental Cost-Effectiveness Ratio (ICER) of the interventions compared to no intervention was measured as cost per averted DALY. Effect sizes and joint effect of interventions used in the analysis were based on systematic reviews of randomized trials and meta-analysis, when possible.

To translate changes in the risk of age and sex specific cardiovascular disease events into changes in population health quantified in terms of DALY, we used a standard methodology described elsewhere [69].

There is no universal criterion that defines a threshold cost-effectiveness ratio, above which an intervention would not be considered cost-effective. We chose to use guidelines specifically intended for international comparisons, as proposed by the Commission on Macroeconomics and Health, which defines interventions with an ICER that is less than three times Gross Domestic Product per capita as a "cost-effective" intervention and as "very cost-effective" if ICER is less than the GDP per capita [70,71]. Argentina's GDP per person in 2007 was estimated in I$ 13,255.09 [72].

### Uncertainty and sensitivity analysis

We also did a probabilistic, multivariate sensitivity analysis using Monte Carlo simulation [73,74] of 1,000 randomly selected sets of variables, to assess the effects of uncertainty in the prevalence of risk factors, population attributable risk and effect sizes of interventions. In addition, an undiscounted scenario was considered for costs and DALY, and a non age-weighted scenario was also analyzed for DALY.

## Results

We estimated a lethality rate of 11.9% in men and 18% in women; and 17.4% in men and 18.9% in women, for CHD and stroke, respectively [17]. According to these estimates, about 263,025 annual acute CHD and stroke events would be expected, representing an annual cost of I$ 1,036,506,958. More than 60% of total events and costs are accounted by men. Table 4 shows the estimation of the overall number of annual cardiovascular events in Argentina, burden of disease and costs of events. As observed, more than 600,000 DALYs and almost 400,000 YPLL were lost in 2005 due to CHD and stroke.

**Table 4:** Estimation of total cases, costs and burden of disease of acute CHD and stroke

	Men	Women	Total
Total AMI events [%]	62,132 [72.1%]	24,031 [27.9%]	86,163 [100.0%]
Total non-infarction events [%]	51,660 [68.5%]	23,751 [31.5%]	75,411 [100.0%]
Total stroke events [%]	53,432 [52.7%]	48,018 [47.3%]	101,450 [100.0%]
Total events [%]	167,225 [63.6%]	95,800 [36.4%]	263,025 [100.0%]
Total costs* I$ [%]	667,728,147 [64.4%]	368,778,811 [35.6%]	1,036,506,958 [100.0%]
Total DALY† [%]	293,419 [48.0%]	318,217 [52.0%]	611,635 [100.0%]
Total PYLL‡ [%]	218,547 [55.5%]	175,617 [44.6%]	394,163 [100.0%]

95%CI: 95% confidence interval, AMI: acute myocardial infarction, DALY: disability-adjusted life years, I$: international dollars, PYLL: Potential Years of Life Lost.

* Only direct medical costs by hospitalization were considered. Costs are measured in 2007 international dollars (I$).

† With discounting (3% annual rate), without age weight.

‡ With discounting (3% annual rate).

### Burden of Disease attributable to modifiable cardiovascular Risk Factors

Population attributable risks, costs of events and DALY lost to cardiovascular disease for the overall risk factors and for each single modifiable risk factor selected, can be seen in Tables 5 and 6, respectively. All risk factors together explained 75% of fatal and non-fatal acute CHD and stroke events, 82,4% of acute CHD events (84.0% in women) and 62.4% of strokes (66,6% in men). Similarly, modifiable risk factors explained 75,5% of costs of acute events and 70.7% of DALY lost. The most important single risk factor was high BP, explaining 37% of all CHD and strokes and one-third of all DALY lost in 2005. The rest of the risk factors have similar attributable burden in term of CV events, ranging between 13,9% (high glycemia) to 18,1% (physical inactivity). (see Table 6).

**Table 5:** Proportional Burden of Disease and costs attributable to all cardiovascular risk factors potentially modifiable

	*Population-attributable Fraction *% (95%CI)		
	Men	Women	Total
Total AMI events (both fatal and non-fatal)	81.8 (81.3-82.2)	84.1 (82.8-85.0)	82.4 (81.9-82.8)
Total non-infarction events (both fatal and non-fatal)	81.5 (80.9-82.0)	83.5 (81.9-84.7)	82.1 (81.5-82.6)
Total stroke events (both fatal and non-fatal)	66.6 (65.7-67.4)	57.6 (56.4-58.8)	62.4 (61.6-63.1)
Total events (both fatal and non-fatal)	76.8 (76.2-77.4)	70.7 (69.4-71.7)	74.6 (74.0-75.1)
Total costs*	77.5 (76.9-78.0)	72.0 (70.7-73.1)	75.5 (75.0-76.0)
Total DALY^†^	76.0 (75.5-76.4)	65.8 (65.0-66.5)	70.7 (70.2-71.1)
Total PYLL^‡^	76.5 (75.9-76.9)	69.6 (68.6-70.5)	73.4 (72.9-73.9)

**Results of 1,000 iterations, both sexes**.

95%CI: 95% confidence interval, AMI: acute myocardial infarction, DALY: disability-adjusted life years, PYLL: Potential Years of Life Lost.

* Only direct medical costs by hospitalization were considered.

† With discounting (3% annual rate), without age weight.

‡ With discounting (3% annual rate).

**Table 6:** Proportional Burden of Disease and costs attributable to each cardiovascular risk factor potentially modifiable

		*Population-attributable Fraction *% (95%CI)				
Both sexes	Tobacco use	Overweight and Obesity	High Blood pressure	High cholesterol	High glycemia	Physical inactivity
Total AMI events (both fatal and non-fatal)	22.5 (22.2 - 22.8)	20.9 (20.4 - 21.3)	38.5 (37.9 - 39.1)	25.1 (24.4 - 25.8)	13.9 (13.2 - 14.5)	20.9 (20.6 - 21.2)
Total non-infarction events (both fatal and non-fatal)	18.5 (18.1 - 18.8)	18.6 (18.2 - 19.0)	40.9 (40.1 - 41.5)	26.2 (25.2 - 27.0)	14.8 (14.0 - 15.6)	21.6 (21.2 - 22.0)
Total stroke events (both fatal and non-fatal)	10.8 (10.5 - 11.2)	11.7 (11.3 - 12.0)	32.7 (32.0 - 33.3)	5.8 (5.6 - 6.1)	13.2 (12.3 - 14.0)	13.0 (12.7 - 13.3)
Total events (both fatal and non-fatal)	16.9 (16.5 - 17.2)	16.7 (16.3 - 17.0)	37.0 (36.3 - 37.5)	18.0 (17.4 - 18.5)	13.9 (13.1 - 14.6)	18.1 (17.7 - 18.3)
Total costs*	17.3 (17.0 - 17.6)	17.1 (16.7 - 17.4)	37.3 (36.6 - 37.9)	18.9 (18.3 - 19.5)	13.9 (13.2 - 14.7)	18.4 (18.1 - 18.7)
Total DALY^†^	16.1 (15.7 - 16.4)	13.8 (13.5 - 14.0)	36.6 (36.1 - 37.0)	13.4 (13.0 - 13.7)	13.6 (13.0 - 14.2)	15.5 (15.2 - 15.7)
Total PYLL^‡^	16.6 (16.3 - 16.9)	15.1 (14.8 - 15.4)	37.5 (36.9 - 38.0)	16.6 (16.1 - 17.1)	13.9 (13.2 - 14.6)	16.9 (16.6 - 17.1)
***Men***	**Tobacco use**	**Overweight and Obesity**	**High Blood pressure**	**High cholesterol**	**High glycemia**	**Physical inactivity**
Total AMI events (both fatal and non-fatal)	26.7 (26.2 - 27.1)	24.7 (24.1 - 25.2)	37.0 (36.4 - 37.6)	19.0 (18.3 - 19.6)	12.4 (11.8 - 13.0)	20.2 (19.8 - 20.6)
Total non-infarction events (both fatal and non-fatal)	23.1 (22.6 - 23.5)	22.3 (21.8 - 22.8)	40.1 (39.3 - 40.8)	19.3 (18.6 - 20.0)	13.5 (12.8 - 14.2)	20.6 (20.2 - 21.2)
Total stroke events (both fatal and non-fatal)	14.2 (13.7 - 14.7)	16.7 (16.0 - 17.3)	35.2 (34.3 - 36.0)	5.2 (4.9 - 5.5)	13.5 (12.6 - 14.4)	12.2 (11.7 - 12.6)
Total events (both fatal and non-fatal)	21.6 (21.1 - 22.0)	21.4 (20.9 - 21.9)	37.4 (36.7 - 38.1)	14.7 (14.1 - 15.2)	13.1 (12.4 - 13.8)	17.8 (17.4 - 18.2)
Total costs*	22.0 (21.6 - 22.5)	21.7 (21.2 - 22.2)	37.6 (36.8 - 38.2)	15.2 (14.7 - 15.8)	13.1 (12.4 - 13.8)	18.1 (17.7 - 18.6)
Total DALY^†^	22.1 (21.6 - 22.6)	20.5 (20.1 - 20.9)	38.7 (38.1 - 39.3)	12.1 (11.7 - 12.6)	13.0 (12.3 - 13.7)	15.6 (15.3 - 16.0)
Total PYLL^‡^	21.6 (21.1 - 22.1)	20.7 (20.2 - 21.1)	38.7 (38.1 - 39.3)	13.4 (12.9 - 13.8)	13.1 (12.4 - 13.8)	16.5 (16.2 - 16.9)
***Women***	**Tobacco use**	**Overweight and Obesity**	**High Blood pressure**	**High cholesterol**	**High glycemia**	**Physical inactivity**
Total AMI events (both fatal and non-fatal)	11.9 (11.5 - 12.3)	11.0 (10.5 - 11.6)	42.4 (41.1 - 43.5)	41.0 (39.2 - 42.7)	17.7 (16.2 - 19.1)	22.6 (22.2 - 23.1)
Total non-infarction events (both fatal and non-fatal)	8.5 (8.0 - 9.0)	10.5 (9.9 - 11.2)	42.5 (40.7 - 44.0)	41.1 (38.4 - 43.1)	17.7 (15.8 - 19.6)	23.7 (23.1 - 24.2)
Total stroke events (both fatal and non-fatal)	7.1 (6.8 - 7.6)	6.1 (5.8 - 6.5)	29.9 (28.9 - 30.8)	6.6 (6.1 - 7.1)	12.8 (11.5 - 14.2)	14.0 (13.6 - 14.3)
Total events (both fatal and non-fatal)	8.6 (8.3 - 9.1)	8.4 (8.0 - 8.9)	36.2 (34.9 - 37.3)	23.8 (22.4 - 24.9)	15.2 (13.8 - 16.8)	18.6 (18.1 - 19.0)
Total costs*	8.8 (8.4 - 9.3)	8.7 (8.2 - 9.7)	36.8 (35.5 - 37.9)	25.5 (24.1 - 26.7)	15.5 (14.0 - 17.0)	19.0 (18.6 - 19.5)
Total DALY^†^	10.5 (10.1 - 10.9)	7.6 (7.3 - 7.9)	34.5 (33.8 - 35.2)	14.5 (13.9 - 15.1)	14.1 (13.2 - 15.1)	15.3 (15.0 - 15.6)
Total PYLL^‡^	10.4 (10.0 - 10.8)	8.3 (7.9 - 8.7)	36.0 (35.0 - 36.9)	20.7 (19.7 - 21.6)	14.8 (13.6 - 16.0)	17.3 (16.9 - 17.6)

**Results of 1000 iterations, basal case, both sexes**.

95%CI: 95% confidence interval, AMI: acute myocardial infarction, DALY: disability-adjusted life years, PYLL: Potential Years of Life Lost.

* Only direct medical costs by hospitalization were considered.

† With discounting (3% annual rate), without age weight.

‡ With discounting (3% annual rate).

### Cost-effectiveness of selected interventions

Table 7 summarizes the results of economic evaluation of the 6 distinct interventions giving their total annual costs and costs per beneficiary, health effects in terms of DALY averted (non age-weighted and 3% discounted), percent of DALY saved due to cardiovascular disease and average cost-effectiveness ratio for each in I$ per DALY averted. Two interventions were cost-saving: lowering salt intake in the population through reducing salt in bread and treatment targeted to persons with an absolute risk above 20% in 10 years (modified polypill strategy). Moreover, the implementation of the polypill strategy was also associated with almost a 2% decrease in DALY lost to cardiovascular disease. On the other hand, the impact of reducing salt in bread was more limited (0.11% of decrease of DALY lost) due in part to the lower extension and magnitude of this intervention. Two interventions had very acceptable ICER: 1) drug therapy for high blood pressure in hypertensive patients not yet under going treatment with an ICER of I$ 2,908 per DALY saved and an annual reduction of 2.3% of cardiovascular disease burden; and 2) mass media campaign to promote tobacco cessation amongst smokers, with an ICER of I$ 3,186 per DALY saved (0.11% of cardiovascular disease burden). An additional intervention, lowering cholesterol with statins (I$ 14,432 per DALY saved), was considered cost-effective according to the guidelines mentioned above. Finally, one intervention, tobacco cessation with bupropion (I$ 59,433 per DALY saved) was not found to be cost-effective. This is in part because bupropion is much more expensive than blood pressure lowering drugs and also because, as it is not currently covered in the public sector, the government does not usually exert its purchasing power to get lower prices. Following local surveys, we assumed that only 11% of the population of smokers would be willing to quit smoking each year and consequently start on a program, the population impact of tobacco cessation therapy was much smaller than expected.

**Table 7:** Cost effectiveness analysis of selected interventions

*Intervention*	*Total costs*	Net Total costs *	*DALY saved †*	*Percent of DALY saved*	*ICER per DALY saved *‡
	I$	I$	DALY (95%CI)	% (95%CI)	I$ (95%CI)
Reducing salt in bread	193,576.23	-946,580.87	672.80	0.11%	**-1,406.93**
	(-)	(-)	(-)	(-)	**(-)**
Treatment targeted to persons with an absolute risk above 20% in 10 years (polypill strategy	23,489,613.55	-2,979,727.10	12,108.15	1.98%	**-246.45**
	(22,002,839.35 - 24,877,996.13)	(-3,663,248.39- -2,301,924.52)	(11,396.45- 12,752.71)	(1.86% - 2.09%)	**(-307.74 - -189.68)**
Pharmacological therapy for high blood pressure	62,251,491.82	41,014,269.81	14,100.16	2.31%	**2,908.86**
	(61,201,512.65 - 63,303,292.70)	(40,070,763.64 - 42,021,481.43)	(13,919.53 - 14,275.31)	(2.28% - 2.33%)	**(2,841.81 - 2,977.48)**
Mass Media Campaign to promote tobacco cessation	3,164,785.75	2,053,674.53	644.73	0.11%	**3,186.71**
	(-)	(2,012,812.78 - 2,090,361.57)	(624.52 - 666.90)	(0.10% - 0.11%)	**(3,024.42 - 3,337.92)**
Pharmacological therapy of high cholesterol	92,751,189.47	81,762,199.07	5,666.16	0.93%	**14,431.46**
	(90,559,448.95 - 94,981,610.94)	(79,775,242.39 - 83,805,632.99)	(5,507.87 - 5,824.03)	(0.90% - 0.95%)	**(14,077.55 - 14,809.92)**
Therapy with Bupropion for	51,778,301.95	50,318,932.00	846.80	0.14%	**59,433.02**
					
tobacco cessation	(50,792,163.89 - 52,752,733.57)	(49,372,843.67 - 51,286,965.18)	(820.26 - 875.93)	(0.13% - 0.14%)	**(57,819.14 - 60,906.25)**

95%CI: 95% confidence interval, I$: international dollars - PPP conversion rate (2007) 1.55 Argentinean peso = 1 I$, DALY: disability adjusted life years, ICER: incremental cost-effectiveness ratio. The ICERs express the results of 1,000 iterations.

* Net Total costs are calculated as Total costs minus the corresponding averted event costs. All costs are measured in 2007 International dollars (I$).

‡ Derived from bootstrapping techniques.

**Figure 1:**
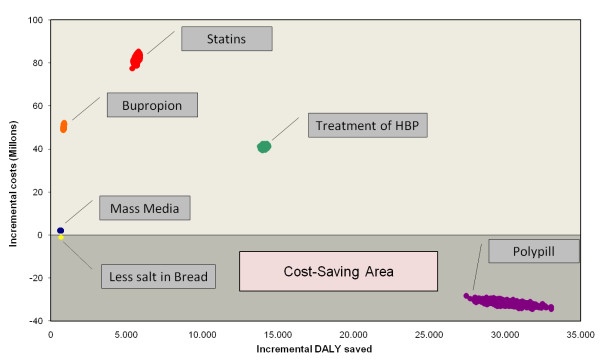
**Interventions along the cost-effectiveness plane**. Costs are expressed in International dollars (I$, 2007).

Figure 1 shows the ICER of the six distinct interventions along the cost effectiveness plane with their respective probability distribution. The shaded area corresponds to the cost-saving interventions.

### Sensitivity Analyses

We examined the effect of a change in the PAR estimate of the overall risk factors selected along a reasonable range of probabilities, by creating alternative scenarios with different prevalence and distributions of risk factors. We also explored undiscounted and age-weighted DALY as compared to the base case scenario with DALY discounted at 3 percent and non age-weighted. In all circumstances, the ranking as well as the magnitude of each intervention remained the same. Finally, the results of the probabilistic sensitivity analyses to estimate the uncertainty surrounding the central estimates of each intervention is expressed through the 95%CI showed in Table 7.

## Discussion

Our study analyzed the FASRF at individual level to estimate the burden of cardiovascular disease in Argentina attributable to modifiable risk factors in order to model the impact of some preventive interventions to reducing this burden, as well as estimating their cost-effectiveness. Based on our data, the PAR of all the risk factors analyzed explained more than 75% of the acute CHD events and strokes in men and women. Only high blood pressure explained more than one-third of the events while each one of the other risk factors explained between 14% and 18%. WHO recently addressed the importance of chronic disease prevention as a neglected health issue in LMIC; achievement of the global goal to reduce chronic disease death rates by 2% every year would avert 36 million deaths between 2005 and 2015 [75,76]. Achieving this target would also save almost 10% of the expected loss in national income in these settings [7]. Considering the growing burden of cardiovascular disease and costs in developing countries, especially for transitional countries like Argentina, this study is critical to provide local decision-makers with information about cardiovascular disease burden. Furthermore, by comparing the relative costs and health effects of interventions for preventing cardiovascular disease, we can focus policy debate concerning the trade-offs or opportunity costs of financing one intervention over another.

Establishing the cost-effectiveness of preventive interventions for cardiovascular disease in developing country contexts is not straightforward, due to both the paucity of existing evidence, and because there is no universally agreed threshold for considering the cost-effectiveness of an intervention to be 'too high' or 'right'. What is acceptable to health and finance decision-makers depends largely on the country context. The Disease Control Priorities Project (DCPP), has identified several chronic disease interventions as cost-effective at a cost of below US$1,000 per DALY [77]. However, the affordability of interventions will vary significantly across countries, even among a group of interventions believed to be cost-effective in the global sense. Moreover, sensitivity analysis done as part of the cost effectiveness analysis modeling for the DCPP showed that the cost-effectiveness of public education campaigns at the population level could be very good or far less favorable depending on how much it cost to reach people using a reasonable range of costs. In addition, even a very inexpensive intervention might not be worth implementing if it targets a chronic disease with low prevalence in a given country or region.

In an earlier analysis, Murray et al. [12] modeled selected population-based and individual health interventions to lower high blood pressure and high cholesterol in the epidemiological contexts of developing countries. The authors found that all interventions were highly cost-effective in the sub-region of the Americas to which Argentina belongs.

More recently, Asaria et al., assessed the financial costs and health effects of a voluntary reduction in the salt content of processed foods by manufacturers plus a mass media campaign to encourage dietary change in 23 selected low and middle income countries, including Argentina. They estimated that a 15% reduction in dietary salt intake in Argentina would save 60,000 lives over the period 2006-2015 at a cost of US$ 0.14 per capita (equivalent to AR$ 16.7 million for a population of Argentina (38 millions in 2005) [38].

As compared to these previous studies [12,38], our intervention to decrease salt intake in bread was cost-saving, although both our health impact and cost estimates were appreciably lower than those summarized above, partly because we only included a series of one-off meetings with bread makers from large cities, and also because we used a lower effect size.

In regards to the intervention oriented to reduce high blood pressure and high cholesterol our ICER were remarkably higher than those reported by Murray et al. [12]. The causes of this apparent discrepancy are two-fold: firstly, the counterfactual scenario designed by Murray, based on the WHO-CHOICE methodology [71] entails lifting the constraints of the current mix of interventions, using a null scenario of no costs and no interventions as a starting point, as opposed to our assumption that almost half of Argentine population were already receiving treatment; based on the data of the FASRF; and secondly, our cost estimates are considerably higher, which reflects the fact that key intervention resource inputs in Argentina (including human resources, secondary care and drugs) are much more expensive than the regional average. The addition of individual-level interventions with a multi-drug regimen on the basis of opportunistic contact with the health service, as reported by Gaziano et al. [78], has been estimated at US$ 2.93 per capita in a country like Argentina, but would save a further 50,000 lives over a 10-year period.

Our analyses have shown that the multidrug regimen of four highly effective drugs (polypill strategy, with an annual cost of I$ 101 or I$ 32 per capita in 2007) could lead to cost-saving prevention and treatment for subjects with an absolute risk above 20% in 10 years, with 2% of reduction in DALY lost to cardiovascular disease even considering a population effectiveness of less than 20% the potential targeted population. Other treatment cutpoints for this intervention, where ICERs would likely be far higher, were not evaluated, including subjects with lesser CV risk or subjects over age 55 as originally proposed by Wald and Law (53), The study of Gaziano et al about the cost-effectiveness of the Polypill regimen [13] modeled an ICER of less than US$ 900 for a similar risk population in the Latin American region.

According to the threshold adopted by World Health Organization, an intervention that saves one DALY for less than three times the gross domestic product (GDP) per capita is considered cost-effective, while one that saves a DALY for less than one GDP per capita is deemed very cost-effective [71]. As Argentina's GDP per person in 2007 was I$13,255 (US$ 6,644) [79,80], estimated ICER of all interventions analyzed except tobacco cessation with bupropion (I$ 59,433 per DALY saved) fell well within the 'very cost-effective' or cost-effective category. In fact, two interventions - reducing salt intake in bread and the absolute risk approach therapy with four drugs - were cost-saving. In an earlier study of cost-effectiveness of cardiovascular interventions in Buenos Aires, in which we used a counterfactual scenario of no costs and no interventions, most of our interventions were very cost-effective [81]. Should we have used a counterfactual scenario based on what the public health sector was actually spending on the care of cardiovascular disease we would have obtained much lower ICER or even cost-saving interventions like we have found in this study.

In addition, the potential budget impact of the implementation of the four cost saving or cost-effective interventions mentioned above was in the range of I$ 194 million in 2005. This expenditure would be partly offset by the savings obtained through avoided cardiovascular acute events. Moreover, the financing of these interventions, even considering low population effectiveness according to our conservative scenario, could reduce at least 7% the cardiovascular disease burden with its consequent health, economic and social impact.

Some limitations of the present work are important to be acknowledged. 1), the risk *factors *included in the model were limited to those that were specifically addressed in the national survey as they were specifically defined. In this regard, concerning the intake of fruits and vegetables, we were bound to the two defined options as posed in the specific question of the survey: more or less than five servings a week (rather than more or less than five servings a day), which is clearly inappropriate based on WHO recommendations [82]. This limitation prompted us to exclude this risk factor for further analysis. Other risk factors related to diet such as trans fat, low marine omega-3, and low polyunsaturated fat were also excluded due to lack of population based data stressing the importance of obtaining future national-level data on these and other dietary risk factors for future analysis; 2) since the prevalence of risk factors was obtained from self-reports of participants and not from direct measures, they were defined dichotomously or categorically (as having or not having the risk factor) for the calculation of the PAR. This implies that the risk of a particular risk factor behaves like an "all or none" phenomenon, which is obviously not true given the continuous nature of this risk in all of the selected risk factors. In this regard, estimating the theoretical minimum risk exposure distribution would be a more appropriate method should this had been possible; 3) we have just modeled interventions that either had been tested in pilot studies (i.e.: reducing salt in bread) or were considered key data to model the intervention (i.e.: just 11% of total smokers in Argentina as potential quitters to model the impact of tobacco cessation with bupropion); 4), we did nor model potential side effects of the multidrug intervention. Ignoring side effects in the analysis could overestimate the ICER of the polypill strategy. 5) our study does not assume any benefit from the pharmacological interventions in the population that is already receiving treatment, as if they were appropriately controlled, which is not true. No matter that this is aligned with our conservative estimates, the ICER of high blood pressure or high cholesterol therapy look less attractive in terms of reduction of disease burden or cost-effectiveness; 6) as in all modeling studies, our study synthesized data from many sources and used several assumptions in the design of the model. As real life decision making tools, these types of model-based studies are explicit analyses to help health priority setting, and are not a "search of the ultimate truth"; and 7) some inputs, as it is also commonplace in modeling studies, were derived from international sources. This was done mainly with relative measures such as relative risks of different cardiovascular risk factors, or relative effects of interventions, which on the other hand, are widely thought to be more generalizable from setting to setting.

## Conclusions

Overall, evidence exists to conclude that there are important clinical as well as economic consequences of cardiovascular disease, consequences that are not only important to the individual and his/her family but also to the economy at large. At the same time, there are severe gaps in the evidence that call for more research into the avoidable burden of cardiovascular disease, in particular for developing countries. Despite the increasing burden of cardiovascular disease in Argentina, ranking first over the last decades as a cause of mortality and morbidity, national health programs and policies are still focused on interventions aimed to tackle communicable diseases or perinatal or childhood conditions, overlooking actions and programs targeted to lifestyle and nutritional changes in the population at large or pharmacological interventions to reduce cardiovascular disease burden in high risk people.

In conclusion, most of the interventions selected were cost-saving or very cost-effective according to WHO standards. Moreover, the financing of these interventions could reduce at least 5% the cardiovascular disease burden with its consequent health, economic and social impact. This study aims to inform policy makers on resource-allocation decisions to reduce the burden of CVD, especially for middle-income developing countries like Argentina.

## Abbreviations

95%CI: 95% confidence interval; AMI: acute myocardial infarction; CEAC: cost-effectiveness acceptability curves; CHD: coronary heart disease; DALY: disability-adjusted life years; DCPP: Disease Control Priorities Project; GDP: Gross Domestic Product; I$: international dollars; ICER: Incremental Cost-Effectiveness Ratio; LMIC: low and middle-income countries; PAF: Population Attributable Fraction; PYLL: Potential Years of Life Lost

## Competing interests

This study was funded by an independent grant from the Comision "Salud Investiga" of the Argentine Ministry of Health (Becas Carrillo-Oñativia).

The authors declare that they have no competing interests.

## Authors' contributions

AR conceived the study, coordinated the teamwork and participated in its design and analysis. He also led the writing of the manuscript. LC carried out the modeling in Excel and Python, and participated in literature search and drafting of the manuscript. AB participated in the literature search, carried out the assessment of measures of effect for conditions and risk factors, and for effectiveness of interventions. AA and KK also participated in the literature search and abstraction of measures of effects. APR and SGM participated in the design of the study and made substantial statistical contributions. LG participated in the modeling design in Python. JC took responsibility for assessing economic disease impacts and costing of the interventions, and helped in the cost effectiveness analysis. FA made substantial contributions in the CEA. All authors read and approved the final manuscript.

## Pre-publication history

The pre-publication history for this paper can be accessed here:

http://www.biomedcentral.com/1471-2458/10/627/prepub
